# Interactions Networks for Primary Heart Sarcomas

**DOI:** 10.3390/cancers13153882

**Published:** 2021-08-01

**Authors:** Styliani A. Geronikolou, Athanasia Pavlopoulou, George P. Chrousos, Dennis V. Cokkinos

**Affiliations:** 1 Clinical, Translational and Experimental Surgery Research Centre, Biomedical Research Foundation Academy of Athens, 4, Soranou Ephessiou Str., 11527 Athens, Greece; chrousge@med.uoa.gr (G.P.C.); dcokkinos@bioacademy.gr (D.V.C.); 2 University Research Institute of Maternal and Child Health & Precision Medicine, National and Kapodistrian University of Athens, Aghia Sophia Children’s Hospital, 11527 Athens, Greece; 3 UNESCO Chair on Adolescent Health Care, National and Kapodistrian University of Athens, Aghia Sophia Children’s Hospital, 11527 Athens, Greece; 4 Izmir Biomedicine and Genome Center (IBG), Balcova, Izmir 35340, Turkey; athanasia.pavlopoulou@ibg.edu.tr; 5 Izmir International Biomedicine and Genome Institute, Balcova, Izmir 35340, Turkey

**Keywords:** heart sarcoma, interactome, personalized medicine, heart failure treatment, primary heart cancer, case report

## Abstract

**Simple Summary:**

Cardiac cancer represents a rare, largely understudied disease. The aim of this study was to elucidate the underlying pathophysiology of cardiac sarcomas by employing specific network-based methods. Focused interactomes comprised of heart- and tumor-associated gene/proteins were constructed. These networks allowed us to unfold the main pathways leading from heart sarcomas to cardiac diseases. The findings of this study could be utilized in the clinical setting for diagnostic and therapy decision-making.

**Abstract:**

Personalized medicine incorporates genetic information into medical practice so as to optimize the management of chronic diseases. In rare diseases, such as heart cancer (incidence 0.0017–0.33%), this may be elusive. Ninety-five percent of the cases are due to secondary involvementwith the neoplasm originating in the lungs, breasts, kidney, blood, or skin. The clinical manifestations of heart tumors (benign or malignant) include heart failure, hypertension, and cardiac arrhythmias of varying severity, frequently resulting in blood vessel emboli, including strokes. This study aims to explain the pathophysiology and contribute to a P4 medicine model for use by cardiologists, pathologists, and oncologists. We created six gene/protein heart-related and tumor-related targets high-confidence interactomes, which unfold the main pathways that may lead to cardiac diseases (heart failure, hypertension, coronary artery disease, arrhythmias), i.e., the sympathetic nervous system, the renin-angiotensin-aldosterone axis and the endothelin pathway, and excludes others, such as the K oxidase or cytochrome P450 pathways. We concluded that heart cancer patients could be affected by beta-adrenergic blockers, ACE inhibitors, QT-prolonging antiarrhythmic drugs, antibiotics, and antipsychotics. Interactomes may elucidate unknown pathways, adding to patient/survivor wellness during/after chemo- and/or radio-therapy.

## 1. Introduction

Since 1999, when Wulff disputed Newton’s mechanistic point of view on ‘disease’, adopting the Aristotelian one (that an organism is a complex of qualities rather than quantities), complexity became an ongoing research subject in medicine and epidemiology [1]. Thus, the term ‘disease’ seems to be redefined by summarizing data from various directions (lifestyle, inherited predispositions, medical history, sensory data, imaging, all -omics) [2]. Noteworthy strides in the field have come from oncology, as well as from cardiology, where complexity and heterogeneity are recognized as dominant features. Most importantly, both entities share the highest morbidity and mortality rates in Western societies, and the ongoing research in these fields strives to elucidate the implicated mechanisms and to search for potential diagnostic and/or therapeutic targets. Generally, cancer patients seldom share the same therapy, even if they are of the same gender, age, education, and lifestyle [3]. The complexity of such an attempt, as well as the treatment drug selection, are subjects of epidemiologic modeling. As the era of precision medicine evolves, epidemiology may profit from systems science, which concerns and encompasses translational research, traditional medicine, and -omics data, necessary to perform precise epidemiologic modeling [2,3,4]. 

Heart cancer is rarely encountered, as its incidence falls between 0.0017–0.33% [5], while heart sarcomas account roughly for one fourth of them [6], according to the Atlas of Tumor Pathology, published by the Armed Forces Institute of Pathology in the United States of America [7,8]. Importantly, cardiac sarcomas (angiosarcoma, rhabdomyosarcoma, leiomyosarcoma, undifferentiated pleomorphic sarcoma, myxofibrosarcoma, synovial sarcoma) are mostly primary cancers. Sarcomas’ prognosis is usually poor” [9], with metastases occurring both early and frequently. Moreover, metastases -relapses or distant-tumors in 45–75% of the cases may be manifested within 15 years, whilst overall survival is 12–17 months after initial diagnosis [10]. 

The clinical manifestations of heart tumors (benign or malignant) may include no or minor symptoms, such as the so-called medically unexplained symptoms (MUS)—including nausea, weight loss, fatigue, fever, dyspnea at rest, etc.—or serious problems, such as heart failure, hypertension, cardiac arrhythmia, peripheral emboli, or strokes. Cardiac sarcomas are mainly asymptomatic until reaching an advanced stage, when, chest pain, dyspnea, congestive heart failure secondary to blood flow obstruction, and systemic responses may be manifested. The relevant clinical manifestations in cardiac cancer are chest palpitations, chest pain (most common), cardiac tamponade (as the pericardium is often involved), and/or syncope [11].

The heart cancer diagnosis is usually made late, as it often starts after a stroke caused by a detached tumor tissue or thrombus. Echocardiography, CT scan and/or MRI are the main diagnostic tools in the clinician’s quiver. Unfortunately, no satisfactory published series of cases are available for the establishment of prognosis and treatment statistics. 

While, chemotherapy is generally preferred for heart metastatic tumors, surgery is suggested in heart sarcomas, even though their underlying biology is still under-investigated. Heart failure induced by heart sarcomas is a major complication that warrants special attention, as, frequently, its underlying mechanisms are relatively unknown. Sparse published information challenges diagnosticians and therapists and begs for education and training. The rarity of the disease suggests personalized management and thoughtful treatment. The genetic profiles are unelucidated. More importantly, the rara avis itself and the location raise ethical issues urging for non-interventional research options. Thus, to explain the pathogenesis of cardiac sarcomas and their manifestations would be of value to cardiologists, pathologists, and oncologists, who normally deal with patients suffering from heart neoplasms. 

## 2. Results

**Figure 1:**
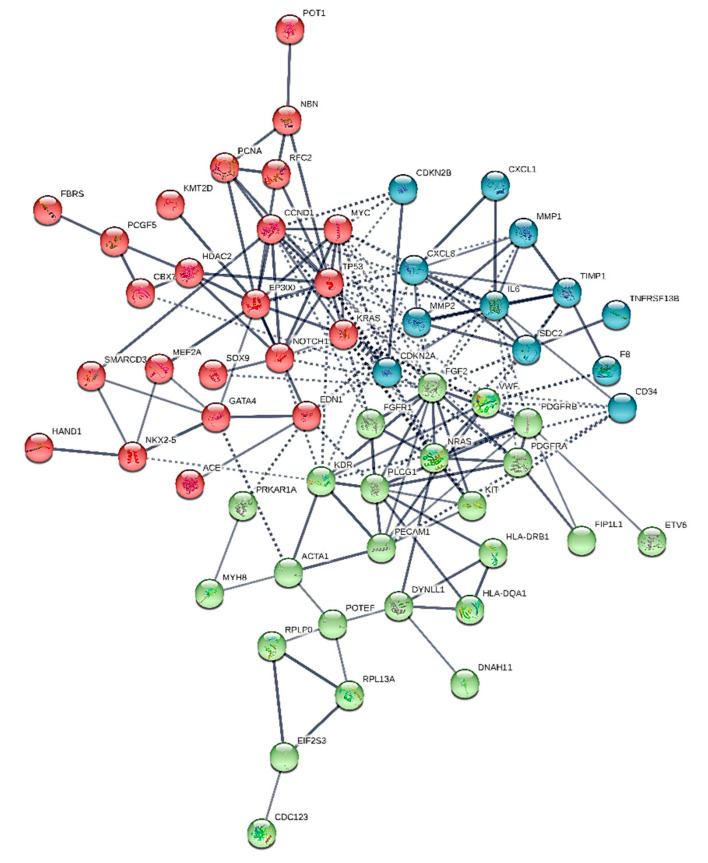
The heart angiosarcoma interactome.

**Figure 2:**
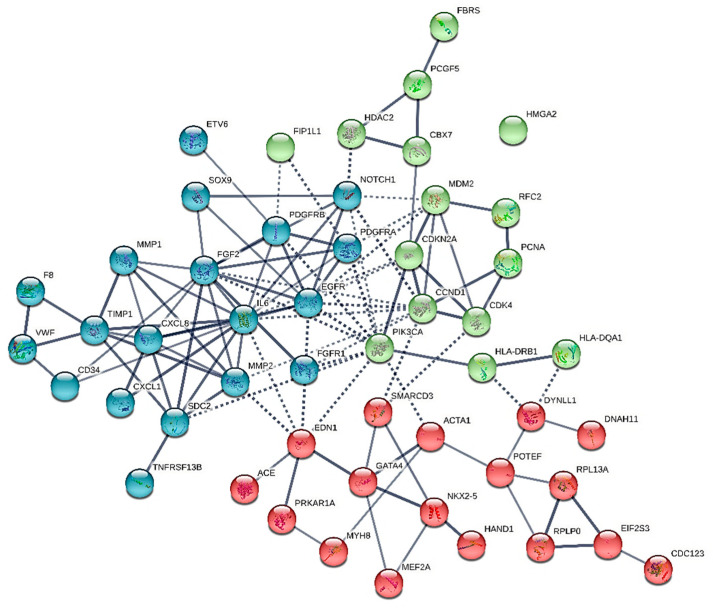
The heart undifferentiated sarcoma interactome.

**Figure 3:**
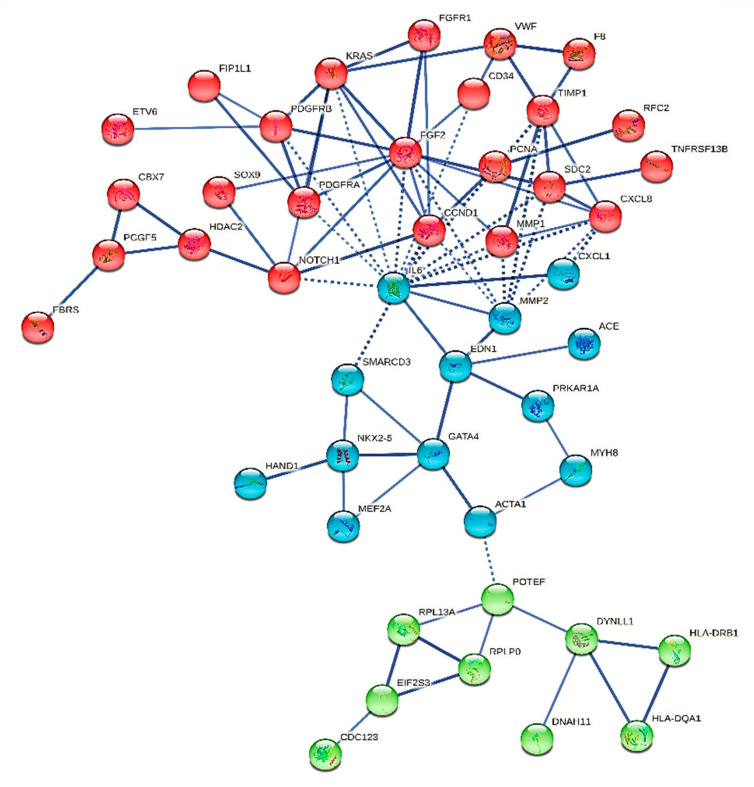
The heart rhabdomyosarcoma interactome.

**Figure 4:**
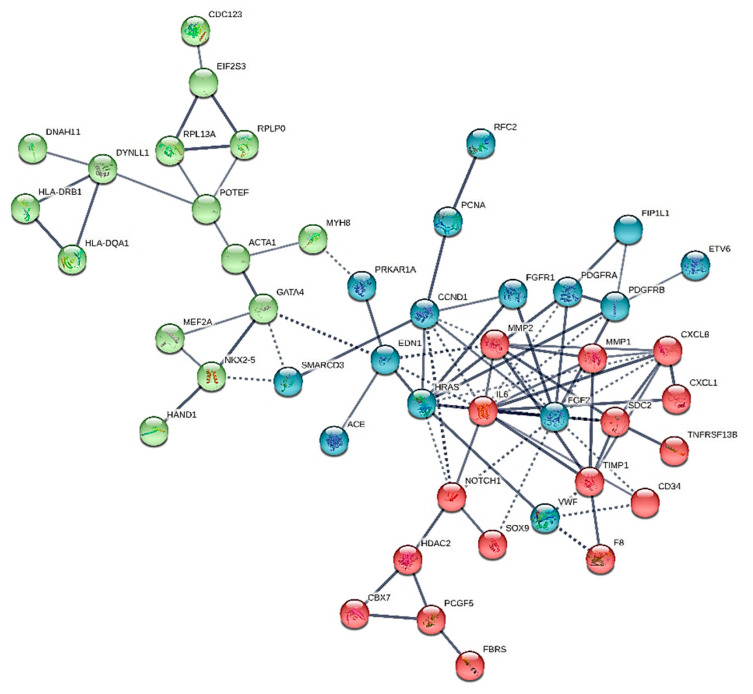
The heart leiomyosarcoma interactome.

**Figure 5:**
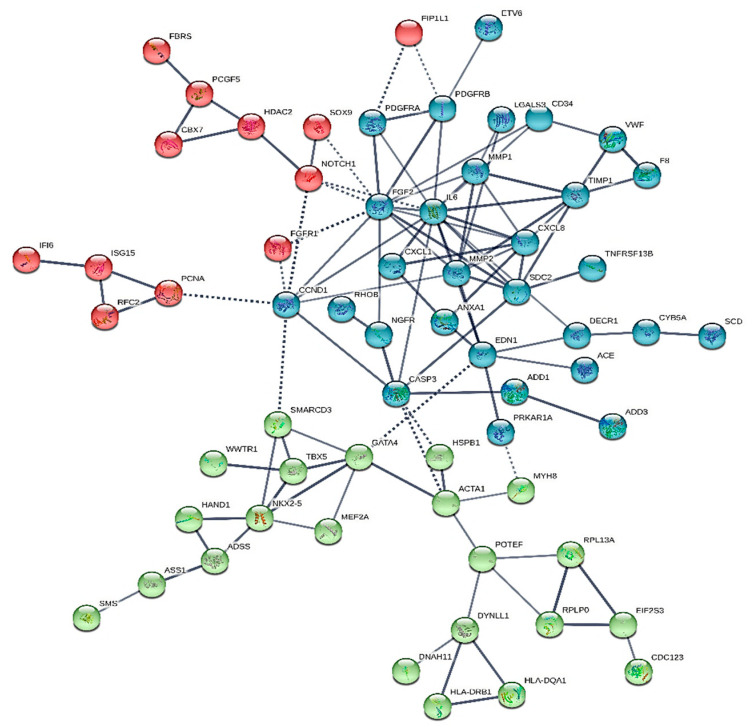
The heart myxofibrosarcoma interactome.

**Figure 6:**
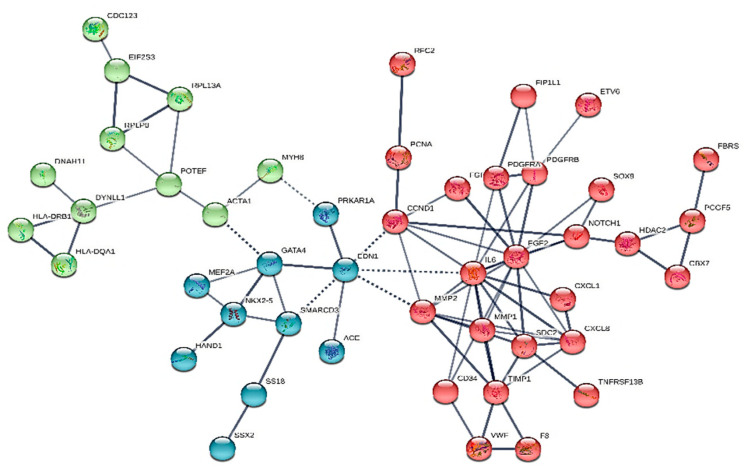
The heart synovial sarcoma interactome.

We constructed six different interaction networks- one for each type of primary heart sarcomas: (i) angiosarcoma, (ii) undifferentiated pleomorphic sarcoma, (iii) Rhabdomyosarcoma (iv) Leiomyosarcoma, (v) Myxofibrosarcoma, and (vi) Synovial sarcoma. The constructed interactomes (henceforth called CS1-6), which include nodes of gene/gene products of known and/or predicted interactions, are described in Table 1 and are illustrated in Figure 1, Figure 2, Figure 3, Figure 4, Figure 5 and Figure 6. A confidence level > 0.7 was adopted. The names of all nodes are shown in the second column of Table 1. The NCBI RefSeq accession numbers [12] are added in a third column, while, in a fourth column, the specific interactome in which each gene is involved, is listed.

### 2.1. Novelties

Eighteen novel intermediate nodes (histone deacetylase 2, cell division cycle 123, fibroblast growth factor 2, polycomb group ring finger 5, syndecan 2, dynein light chain LC8-type 1, POTE ankyrin domain family member F, proliferating cell nuclear antigen, platelet and endothelial cell adhesion molecule 1, nibrin, nerve growth factor receptor, adducin 1, adenylosuccinate synthase 2, cyclin dependent kinase 4, caspase 3, ISG15 ubiquitin like modifier, ISG15 ubiquitin like modifier, high mobility group AT-hook 2, E1A binding protein p300, 2,4-dienoyl-CoA reductase 1, E1A binding protein p300), are distributed in the six networks. 

### 2.2. Major Hubs

In total, 18 highly connected nodes (cyclin D1, endothelin 1, matrix metallopeptidase 2, fibroblast growth factor 2, interleukin 8 (CXCL8), interleukin 6, G protein subunit beta 3,) were revealed.

## 3. Discussion

Heart failure, coronary artery disease, and hypertension epidemics are major public health problems of high prevalence, whose pathophysiology has been insufficiently examined, especially when implicated in other diseases [13]. Heart cancer, on the other hand, is a rare neoplastic entity, that diagnosticians cannot decipher or evenly aptly treat, due to reasonable lack of familiarity [8]. The relevant research demands considerable costs and its effectiveness is time- and population size-dependent. Hence, in silico analysis as gene/gene products interactions networking (interactome construction) is a sine qua non cost/time-effective way to fill in gaps in knowledge in the little known and highly complex field of heart cancer and its cardiological consequences [4]. The latter include heart failure, hypertension, and cardiac arrhythmias, leading to peripheral emboli or even strokes. 

In a previous work, we had constructed the heart cancer interactions network (HFCC1) [14,15]. This work is an expansion of that preliminary work (HFCC1), as it is more focused (on sarcomas) and more specialized. It involves six different interaction networks, one for each heart sarcoma (CS 1–6) (Table 1). 

Our constructed interactomes involve genes with their products that been implicated in various pathological mechanisms:

- oncogenesis (i.e., cyclin D1, CBX7, ETV6, KHDC3L, LINC00457, FGFR1, MMP1, and GRAMD1B) [16,17,18,19,20,21]
- heart failure (i.e., VWF, ACE2, EDN1, PDGFRB, GATA4, MEF2A, NOS3) [22,23,24]
- ischemia (NOS3, SCN5A) [23]
- hypertension (i.e., ACE2, GNB3, EDN1, NOS3) [22,23,25]
- atrial fibrillation (i.e., GATA4, CD34) [26,27]
- atherogenesis (i.e., NOTCH1, F8, NOS3) [23,28]
- inflammation (i.e., IL6, IL8, CD34, VWF, HLA-DQA1),
- oxidative stress (i.e., MMP2) [29]
- renin angiotensin-aldosterone system (i.e., ACE) [22]
- endothelin system (EDN1) [25]
- α-adrenergic signaling (i.e., GBNB3) [30]

Additional physiology mechanisms are also involved, influencing:

- cell survival (i.e., FGF2) [31]
- homeostasis (i.e., ACE2, GRAMD1B) [22]
- hemostasis (i.e., VWF) [32]
- hematopoiesis (i.e., CD34) [33]
- endocrine (i.e., autocrine CXCL1) [34]
- metabolism (i.e., GRAMD1B) [35]
- autonomic nervous system (i.e., ACE2, VWF, MMP2) [22,29]

More specifically, genes known [9,36,37] to be associated with each type of heart sarcomas are shown below:

(i) Angiosarcoma: (POT1, CDKN2A/B, PLCG1, KIT and KDR, MYC, TP53, KMT2D, NRAS or KRAS)
(ii) Undiferrentiated pleomorphic sarcoma: (PDGFRB, FH and PIK3CA mutations KIT, PDGFRA/b, EGFR and MDM2 amplifications, CDKN2A deletion)
(iii) Rhabdomyosarcoma: (KRAS)
(iv) Leiomyosarcoma: (HRAS mutation)
(v) Myxofibrosarcoma: (IFI6, LGALS3, ANXA1 and ASS1 downregulation CYB5A, SCD, ADD3, HSPB1, SMS, WWTR1 and RHOB upregulation)
(vi) Synovial sarcoma: (SS18-SSX fusion)

The highly connected nodes (hubs) are cyclin D1, endothelin 1 (endothelin pathway), matrix metallopeptidase 2 (implicated in protein or RNA binding), fibroblast growth factor 2 (cell survival), interleukin-8, interleukin-6 (inflammation marker), G protein subunit beta 3 (involved in α-adrenergic signaling), nitric oxide synthase 3 (involved in cardiovascular disorders and hypertension). 

Seventeen novel genes were detected:

1. histone deacetylase 2-implicated in tumorigenesis (HDAC2), chronic obstructive pulmonary disease, lung diseases [38]
2. fibroblast growth factor 2 (FGF2) implicated in cell survival and oncogenesis [38]
3. polycomb group ring finger 5 that is involved in oncogenesis [39]
4. syndecan 2 that has been associated to non-alcoholic fatty liver, hepatic fibrosis, post-traumatic stress disorder
5. dynein light chain LC8-type 1 that is implicated in prostate carcinoma in situ [39,40]
6. POTE ankyrin domain family member F, that has been related to dilated cardiomyopathy [41].
7. adducin 1 implicated in the cell volume homeostasis, cell morphogenesis and cellular response to calcium ions [42]
8. adenylosuccinate synthase 2 that is implicated in the de novo pathway and in the salvage pathway of purine nucleotide biosynthesis, it catalyzes the first committed step in the biosynthesis of AMP from IMP
9. caspase 3, a canonical pro-apoptotic protein [43]
10. cyclin dependent kinase 4—a cell division protein encoded by the CDK4 gene—which is associated to tumorigenesis of variant cancers [44]
11. 2,4-dienoyl-CoA reductase 1 protein encoded by DECR1 gene participating in the beta-oxidation and metabolism of unsaturated fatty enoyl-CoA esters [45]
12. E1A binding protein p300, associated to various syndromes and epithelial cancers [46]
13. high mobility group AT-hook 2 which is a transcription factor related to malignancy and poor prognosis
14. ISG15 ubiquitin like modifier—a small ribosomal subunit is a multi-modal unit implicated to immune response and more interestingly to cancer stem cells in a tumor [47]
15. nibrin, a protein involved in DNA damage repair [48], whilst its variants were shown to be associated with breast cancer [49]
16. nerve growth factor receptor—a neurotrophic factor involved in the regulation and survival of certain neurons and pancreatic beta cells [50]
17. platelet and endothelial cell adhesion molecule 1—a protein that removes aged neutrophils from the body, involving in leucocyte transmigration and angiogenesis [51].

Four non-mediating genes were recognized, namely: GRAM domain containing 1B (involved in cholesterol metabolism and oncogenesis), KH domain containing 3 like (involved in oncogenesis), subcortical maternal complex member (implicated in maternal effect of unknown exact function), unc-51 like kinase 4 (associated to bipolar disorder and schizophrenia).

The networks include oncogenes (i.e., cyclin D1), genes (i.e., EDN1, FGF2), enzymes (i.e., MMP2, HDAC2), ion channels (i.e., SCN5A), transcription factors (i.e., SOX9), proteins (i.e., IL-6, IL-8, POTE ankyrin domain family member F, G protein subunit beta 3), cancer cell apoptosis modulator (RHOB). 

The created interactomes elucidate the main pathways leading to cardiac diseases (heart failure, hypertension, coronary artery disease, arrhythmias), such as the renin–angiotensin–aldosterone system (RAAS) and endothelin system, and excludes others, such as K oxidase or cytochrome P450 pathways in these patients. [52,53,54,55]: Based on the Mestroni et al. pharmacogenetics study, we noted that only EDN1 and ACE are included in the created interactomes. Although our study is preliminary, this finding is important for personalized medicine, but still needs to be validated in clinical settings in the future.

**Figure 7:**
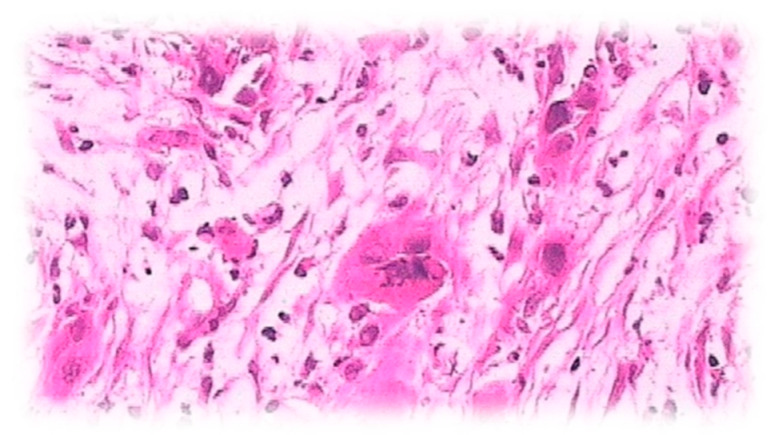
ED3: a representative case of angiosarcoma of the right atrium in a 40-year-old woman: blood vessels are covered by atypical endothelial cells and surrounded by spindle cells.

Angiosarcomas represent 40% of the cardiac sarcomas, are usually found in the right atrioventricular groove (Figure 7) [56], and often expand to the right atrial wall and pericardium. According to the 2015 WHO classification of tumors of the heart and pericardium, angiosarcomas ICD-O code is 9120/3 [56]. The mean prevalent age of heart sarcomas is 41 years, with angiosarcomas accounting for 37% of the total cardiac sarcomas.

The constructed interactome (henceforth called CS1) illustrated in Figure 1 involves 61 nodes. Its major hubs are IL-6, FGF2, TP53, KRAS, CCND1. The following genes are expressed exclusively in angiosarcomas and not in the rest sarcoma types: CDKN2A, CDKN2B, EP300, KIT, NRAS, POT1, NBN, KDR, KMT2D, PPLG1. Our study confirms the findings of literature references [57]. Notably, KRAS is expressed in angiosarcoma and rhabdomyosarcoma as well. 

Connections coming through CXCL1, MMP1, TIMP1, CXCL8 connect to IL-6 and subsequently drive to SDC2, FGF2, CDKNA2, TP53. TP53 through MYC connects to KRAS. The latter connects to EP300 and KMTD2 and through CDKNA2 to FGF2 and NRAS, KIT, PLCG1, and KDR.

We revealed novel not previously reported interactions in CS 1: platelet and endothelial cell adhesion molecule 1 (PECAM1) and nibrin (NBN). 

Moreover, we identified one case of this extremely rara avis cancer entity (angiosarcoma) in the Onassis Cardiac Surgery Center in Athens, which is presented for the first time (Figure 7): a Greek woman < 41 years of age within the established in literature age range. Of note, our in silico study is not based on the individual data of this case. 

The undifferentiated pleomorphic sarcoma is classified with the code number 8830/3 in the WHO classification of tumors of the heart and pericardium of 2015 [56].

The relevant interactome (as of now called CS2) is presented in Figure 2. CS2 consists of 51 nodes, of which IL-6, FGF2, EGFR, and CCND1 are the most interactant molecules (hubs). Five genes are expressed only in this type of sarcoma: PIK3CA, MDM2, HMGA2, EGFR, CDK, CDK4, while CDKN2A is implicated in angiosarcomas and undifferentiated pleomorphic sarcomas only (Table 1, Figure 1 and Figure 2). In this subtype of sarcoma, CDKN2A firmly connects to MDM2, CCND1, PIK3CA, CDK4, CBX7, and less firmly to EGFR, and IL-6. More importantly, IL-6, FGF2, and PDGFRB form a triangle through which they connect to MDM2, CDKN2A and PIK3CA, CCND1, and CDK4, while they connect to PIK3CA, EDN1, ACE, etc. through PDGFRA, and EGFR. 

Rhabdomyosarcoma classification number in the ICD-O Classification of Diseases for Oncology of 2015 is 8900/3 [56]. It is a tumor type routinely found in infants even fetuses, thus, assumed to be a congenital hamartoma. No sexual dimorphism has been reported, while it is usually localized in the ventricular myocardium or every so often project into the heart cavity. Homogenous echogenicity characterizes imaging of these tumors, whilst they incite arrhythmias. The rhabdomyosarcoma (hence called CS3) interactions network numbers 46 nodes, where IL-6 and FGF2 are its most connected nodes. CS3 is described in Figure 3. KRAS is involved both in angiosarcomas and rhabdomyosarcomas. It straightforward connects to PDGFR13, PDGFRA, FGF2, FGFR1, CCND1, and IL-6.

Leiomyosarcoma ICD-O code number in the ICD-O Classification of Diseases for Oncology of 2015 is 8890/3 [56]. This type of sarcoma is typically found in the left atrium, and has been characterized by specific tissue differentiation. The relevant interactome subsequently referred to as CS4 is shown in Figure 4 and contains only 46 nodes. It is the smallest network constructed herein, whilst its major hubs are IL-6, FGF2, and HRAS. The latter is expressed exclusively in this type of heart sarcoma, and, thus, may be assumed as typical of the entity. FGF2 interacts directly with IL-6 that interacts in its turn with HRAS. The latter connects directly to EDN1 or CCND1, FGFR1, MMP2, and VWF. 

Myxofibrosarcoma is coded with the number 8811/3 in the ICD-O Classification of Diseases for Oncology of 2015 [56]. It was previously called malignant fibrous histiocytoma with at least one-quarter of the myxoid areas representing most of the left atrial sarcomas. The network created is henceforth called CS5 (Figure 5) and involves 62 nodes, while it is the most crowded network presented herein. Its major hubs are IL-6, FGF2, and MMP2. 

This network is the only one that involves RHOB- a cancer cell apoptosis modulator and, currently a research target as a cancer therapeutic. As apoptotic factor, it decreases while tumors proliferate, whilst it has been identified in head and neck, lung, and brain cancers and in adenocarcinomas, as a poor prognosis indicator. 

HSPB1, IFI6, WWTR1, SMS, NGFR, LGALS3, ISG15, SCD, CYB5A, DECR1, ADD1, ADD3, ADSS, ANXA1, ASS1, CASP3 are also expressed exclusively in CS 5. ADD1 and ADD3 are interconnected with CASP3 their only node. CASP3, in its turn, connects directly to IL-6, NGFR, SDC2, HSB1, ACTA1, and CCND1. DECR1 connects directly to CYB5A, EDN1, SDC2. NGFR interacts with FGF2 and RHOB only. ANXA1, in its turn, has three connections: EDN1, SDC2, CXCL8. LGALS3 interacts only with MMP1, MMP2. CYB5A is linked to SCD, except DECR1. ISG15 and IF16 are directly linked together but through PCNA and then through CCND1 they connect indirectly to the common to all sarcomas’ major hubs FGF2 and IL-6. SMS connects to ASS1 which connects to ADSS and then to NKX2-5, which through GATA4 affects EDN1. 

In keeping with the ICD-O Classification of Diseases for Oncology of 2015, the synovial sarcoma’s code number is 9040/3 [56]. The interactions network we have built (henceforward called CS6) includes 47 nodes and is shown in Figure 6. The high degree connections of this specific interactome are IL-6 and FGF2. Notably, HRAS, SS18, SSX2, MVP2 are uniquely expressed in synovial heart sarcoma. Of those, SSX2 and SS18 have been identified in histology-type investigations in the literature, whilst MVP2 function is little known, while HRAS is implicated in various types of cancers (i.e., salivary duct carcinoma [58], epithelial myoepithelial carcinoma [59], etc.

In sum, we observed that all cardiac sarcomas share

- forty-four common nodes
- two common hubs: IL-6 and FGF2, that may be assumed as typical of the overall entity (heart sarcomas)

All six interactions networks are presented in 3 basic color-specified units/clusters. Nodes in a given cluster are most closely connected to those nodes of the corresponding cluster, as compared to the other two clusters. Nodes in the same cluster module can be associated with similar/common biological functions. This information could be expanded to more complicated enriched clusters that represent cardiac sarcoma-related biological processes, in the future. 

Finally, the wild-type gene inclusion is a limitation in this investigation.

## 4. Materials and Methods

**Figure 8:**
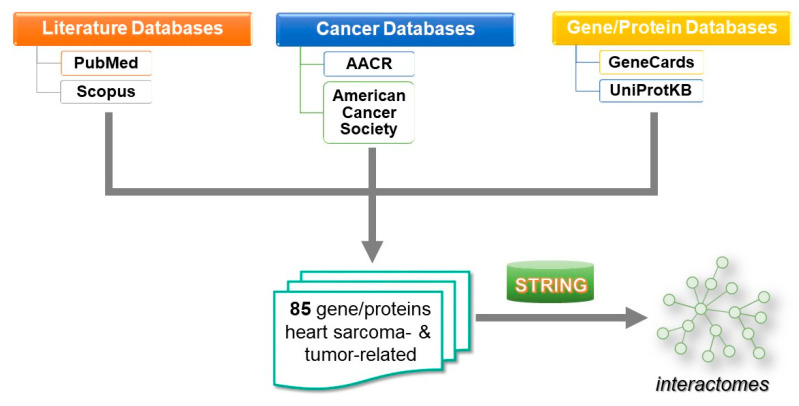
Workflow chart representing the algorithm followed in this work.

The protocol followed is the established one in molecular networking [60,61] and is described in Figure 8. 

An extensive search in literature (PUBMED, Scopus), databases (GeneCards, UniProt) and cancer official sites (https://www.aacr.org/, accessed on 31 March 2021, https://www.cancer.org/, accessed on 31 March 2021), was conducted in the period December 2018–January 2021, revealing 85 gene/protein heart sarcomas-related and tumor-related targets. The interactions among gene/gene products were studied through STRING v10 [62]. A relative high confidence interaction score of 0.7–0.97 was opted so as to extract relevant information from multiple and diverse sources, avoiding at the same time the inclusion of rather erroneous interactions (e.g., false positives). The average degree of connectivity (i.e., mean number of connections of a given node to its immediate neighbors) and *k*-means clustering in the constructed networks were computed with the usage of algorithms implemented in the STRING database. The nodes in the network were partitioned into three clusters, based on the shortest path (distance) between two given nodes. Intra-cluster edges are represented by solid lines, whereas inter-cluster edges are denoted by dashed lines. 

Genes not previously published to be associated to the specific heart sarcoma type, thus, not been included in the initial set of molecules in String platform so as to be processed are described as ‘novel’ or ‘novelties’ in this text.

All six interaction networks are presented in three basic color-specified units/clusters. Nodes in a given cluster are most closely connected to those nodes of the corresponding cluster, as compared to the other two clusters. Nodes in the same cluster module can be associated with similar/common biological functions.

## 5. Conclusions

Cardiac sarcoma patients might profit from administration of beta-adrenergic blockers, ACE inhibitors, QT-prolonging antiarrhythmic drugs, antibiotics, and antipsychotics. Interactomes may elucidate unknown cardiac implications of cardiac malignancies and contribute to patient/survivors’ wellness during and after chemo and/or radio treatment. 

## Figures and Tables

**Table 1:** Molecules included in the primary heart sarcomas interactions networks CS 1–6.

Gene Symbol	Description	Accession Number ^1^	Interactome ^2^
ACE	angiotensin I converting enzyme	NM_000789	CS 1–6
ACTA1	actin alpha 1, skeletal muscle	NM_001100	CS 1–6
ADD1	adducin 1	NM_014189	CS 5
ADD3	adducin 3	NM_019903	CS 5
ADSS	adenylosuccinate synthase 2	NM_001126	CS 5
ANXA1	annexin A1	NM_000700	CS 5
ASS1	argininosuccinate synthase 1	NM_000050	CS 5
CASP3	caspase 3	NM_004346	CS 5
CBX7	chromobox 7	NM_175709.5	CS 1–6
CCND1	cyclin D1	NM_053056.3	CS 1–6
CD34	CD34 molecule	NM_001773	CS 1–6
CDC123	cell division cycle 123	NM_006023	CS 1–6
CDK4	cyclin dependent kinase 4	NM_000075	CS 2
CDKN2A	Cyclin Dependent Kinase Inhibitor 2A	NM_001025109	CS 1, 2
CDKN2B	Cyclin Dependent Kinase Inhibitor 2B	NM_006023	CS 1
CXCL1	C-X-C motif chemokine ligand 1	NM_001511	CS 1–6
CXCL8	C-X-C motif chemokine ligand 8	NM_000584	CS 1–6
CYB5A	cytochrome b5 type A	NM_001914	CS 5
DECR1	2,4-dienoyl-CoA reductase 1	NM_001330575	CS 5
DNAH11	dynein axonemal heavy chain 11	NM_001277115	CS 1–6
DYNLL1	dynein light chain LC8-type 1	NM_001037494	CS 1–6
EDN1	endothelin 1	NM_001955	CS 1–6
EIF2S3	eukaryotic translation initiation factor 2 subunit gamma	NM_001415	CS 1–6
EGFR	epidermal growth factor receptor	NM_005228	CS 2
EP300	E1A binding protein p300	NM_001429	CS 1
ETV6	ETS variant 6	NM_001987	CS 1–6
F8	coagulation factor VIII	NM_000132	CS 1–6
FBRS	Fibrosin	NM_001105079	CS 1–6
FGF2	fibroblast growth factor 2	NM_001361665	CS1–6
FGFR1	fibroblast growth factor receptor 1	NM_023110	CS1–6
FIP1L1	factor interacting with PAPOLA and CPSF1	NM_030917	CS1–6
GATA4	GATA binding protein 4	NM_001308093	CS 1–6
HAND1	heart and neural crest derivatives expressed 1	NM_004821	CS 1–6
HDAC2	histone deacetylase 2	NM_001527	CS 1–6
HLA-DQA1	major histocompatibility complex, class II, DQ alpha 1	NM_002122	CS 1–6
HLA-DRB1	major histocompatibility complex, class II, DR beta 1	NM_002124.4	CS 1–6
HMGA2	high mobility group AT-hook 2	NM_003483	CS 2
HRAS	HRas proto-oncogene, GTPase	NM_176795	CS 4
HSPB1	heat shock protein family B (small) member 1	NM_001540	CS 5
IFI6	interferon alpha inducible protein 6	NM_022873	CS 5
IL6	interleukin 6	NM_000600	CS 1–6
ISG15	ISG15 ubiquitin like modifier	NM_005101	CS 5
KDR	kinase insert domain receptor	NM_002253	CS 1
KIT	KIT proto-oncogene, receptor tyrosine kinase	NM_000222	CS 1
KMT2D	lysine methyltransferase 2D	NM_003482	CS 1
KRAS	KRAS proto-oncogene, GTPase	NM_033360	CS 1, CS 3
LGALS3	galectin 3	NM_002306	CS 5
MDM2	MDM2 proto-oncogene	NM_002392.6	CS 2
MEF2A	myocyte enhancer factor 2A	NM_001130926	CS 1–6
MMP1	matrix metallopeptidase 1	NM_002421	CS 1–6
MMP2	matrix metallopeptidase 2	NM_001127891	CS 1–6
MYC	MYC proto-oncogene, bHLH transcription factor	NM_001354870	CS 1
MYH8	myosin heavy chain 8	NM_002472	CS 1–6
NBN	nibrin	NM_001024688	CS 1
NGFR	nerve growth factor receptor	NM_002507	CS 5
NKX2-5	NK2 homeobox 5	NM_001166175	CS 1–6
NOTCH1	notch 1	NM_017617	CS 1–6
NRAS	NRAS Proto-Oncogene, GTPase	NM_002524.5	CS 1
PCGF5	polycomb group ring finger 5	NM_032373	CS 1–6
PCNA	proliferating cell nuclear antigen	NM_002592	CS 1–6
PDGFRA	platelet derived growth factor receptor alpha	NM_006206	CS 1–6
PDGFRB	platelet derived growth factor receptor beta	NM_002609	CS 1–6
PECAM1	platelet and endothelial cell adhesion molecule 1	NM_000442	CS 1
PIK3CA	phosphatidylinositol-4,5-bisphosphate 3-kinase catalytic subunit alpha	NM_006218	CS 2
PLCG1	phospholipase C gamma 1	NM_182811	CS 1
POT1	protection of telomeres 1	NM_001042594	CS 1
POTEF	POTE ankyrin domain family member F	NM_001099771	CS 1–6
PRKAR1A	protein kinase cAMP-dependent type I regulatory subunit alpha	NM_001276289	CS 1–6
RHOB	ras homolog family member B	NM_004040	CS 5
RFC2	replication factor C subunit 2	NM_181471	CS 1–6
RPL13A	ribosomal protein L13a	NM_001270491	CS 1–6
RPLP0	ribosomal protein lateral stalk subunit P0	NM_053275	CS 1–6
SCD	stearoyl-CoA desaturase	NM_005063	CS 5
SDC2	syndecan 2	NM_002998	CS 1–6
SMARCD3	SWI/SNF related, matrix associated, actin dependent regulator of chromatin, subfamily d, member 3	NM_001003801	CS 1–6
SMS	spermine synthase	NM_004595	CS 5
SOX9	SRY-box 9	NM_000346	CS 1–6
SS18	SS18 subunit of BAF chromatin remodeling complex	NM_001007559	CS 6
SSX2	SSX family member 2	NM_003147	CS 6
TIMP1	TIMP metallopeptidase inhibitor 1	NM_003254	CS 1–6
TNFRSF13B	TNF receptor superfamily member 13B	NM_012452	CS 1–6
TP53	tumor protein p53	NM_000546	CS 1
VWF	von Willebrand factor	NM_000552	CS 1–6
WWTR1	WW domain containing transcription regulator 1	NM_015472	CS 5

^1^ NCBI’s RefSeq (https://www.ncbi.nlm.nih.gov/refseq/, accessed on 31 March 2021); ^2^ CS1: Angiosarcoma; CS2: Undifferentiated pleomorphic sarcoma; CS3: Rhavdomyosarcoma; CS4: Leiomyosarcoma; CS5: Myxofibrosarcoma; and CS6: Synovial sarcoma

## Data Availability

No new data were created or analyzed in this study. Data sharing is not applicable to this article.
